# Intrinsic Resistance of Chronic Lymphocytic Leukemia Cells to NK Cell-Mediated Lysis Can Be Overcome *In Vitro* by Pharmacological Inhibition of Cdc42-Induced Actin Cytoskeleton Remodeling

**DOI:** 10.3389/fimmu.2021.619069

**Published:** 2021-05-24

**Authors:** Hannah Wurzer, Liza Filali, Céline Hoffmann, Max Krecke, Andrea Michela Biolato, Jérôme Mastio, Sigrid De Wilde, Jean Hugues François, Anne Largeot, Guy Berchem, Jérôme Paggetti, Etienne Moussay, Clément Thomas

**Affiliations:** ^1^ Cytoskeleton and Cancer Progression, Department of Oncology, Luxembourg Institute of Health, Luxembourg City, Luxembourg; ^2^ Faculty of Science, Technology and Medicine, University of Luxembourg, Esch-sur-Alzette, Luxembourg; ^3^ Department of Hemato-Oncology, Central Hospitalier du Luxembourg, Luxembourg City, Luxembourg; ^4^ Laboratory of Hematology, Centre Hospitalier de Luxembourg, Luxembourg City, Luxembourg; ^5^ Tumor-Stroma Interactions, Department of Oncology, Luxembourg Institute of Health, Luxembourg City, Luxembourg; ^6^ Department of Oncology, Luxembourg Institute of Health, Luxembourg City, Luxembourg

**Keywords:** actin cytoskeleton, Cdc42, immune evasion, immunological synapse, tumor immunology, natural killer (NK), B cell neoplasms

## Abstract

Natural killer (NK) cells are innate effector lymphocytes with strong antitumor effects against hematologic malignancies such as chronic lymphocytic leukemia (CLL). However, NK cells fail to control CLL progression on the long term. For effective lysis of their targets, NK cells use a specific cell-cell interface, known as the immunological synapse (IS), whose assembly and effector function critically rely on dynamic cytoskeletal changes in NK cells. Here we explored the role of CLL cell actin cytoskeleton during NK cell attack. We found that CLL cells can undergo fast actin cytoskeleton remodeling which is characterized by a NK cell contact-induced accumulation of actin filaments at the IS. Such polarization of the actin cytoskeleton was strongly associated with resistance against NK cell-mediated cytotoxicity and reduced amounts of the cell-death inducing molecule granzyme B in target CLL cells. Selective pharmacological targeting of the key actin regulator Cdc42 abrogated the capacity of CLL cells to reorganize their actin cytoskeleton during NK cell attack, increased levels of transferred granzyme B and restored CLL cell susceptibility to NK cell cytotoxicity. This resistance mechanism was confirmed in primary CLL cells from patients. In addition, pharmacological inhibition of actin dynamics in combination with blocking antibodies increased conjugation frequency and improved CLL cell elimination by NK cells. Together our results highlight the critical role of CLL cell actin cytoskeleton in driving resistance against NK cell cytotoxicity and provide new potential therapeutic point of intervention to target CLL immune escape.

## Introduction

Chronic lymphocytic leukemia (CLL) is the most prevalent lymphoproliferative disorder in the United States and Europe and is characterized by the clonal expansion of mature CD5**^+^** CD23**^+^** B cells  (1, 2). As first reported in 1999, the mutational status of the immunoglobulin heavy chain variable region genes (*IGHV*) is associated with overall survival and is by now considered one of the most important molecular prognostic factors (3, 4). A higher degree of somatic mutations is considered a good prognostic marker, while patients with a non-mutated IgH_V_ region show shorter progression-free and overall survivals (5). An additional marker of prognostic and predictive value is the tumor protein 53 gene (*TP53*) status that can be affected by 17p13 deletion ((del(17p)) and/or somatic *TP53* mutations (6, 7). The double knockout of the *TP53* gene renders CLL cells resistant to most chemo-immunotherapies and has also been shown to be involved in resistance against monoclonal antibodies, such as rituximab (8).

A cardinal feature of CLL is an acquired immune system dysregulation and immune response dysfunction of both innate and adaptive immunity that gradually worsens over time even without disease progression (9–19). The immune response of cytotoxic lymphocytes, such as cytotoxic CD8^+^ T and (natural killer) NK cells, is regulated by molecular interactions occurring in the context of immunological synapses (IS). The formation of a lytic IS between cytotoxic lymphocytes and their target cells is a tightly coordinated process that ensures that only infected or transformed cells are lysed (20–22). Because the IS is the point of convergence for cytolytic effector functions, it is susceptible to immune evasion strategies of cancer (23, 24). Interestingly, it has been shown that immune escape of CLL can be achieved by various means, notably by those interfering with the formation or the function of the lytic IS (25–29). Lytic IS formation of CD8^+^ T cells and NK cells with CLL cells can be rescued in part by drug treatments, such as lenalidomide or blocking antibodies, or can even be bypassed by infusion of genetically modified cytotoxic lymphocytes which do not rely on MHC-mediated antigen presentation as they form a non-classical IS (30–36). Nevertheless, toxic side effects or acquired resistance against these new therapeutic options have been reported and result in disease progression  (37, 38).

NK cells are commonly described to play an important role in the immunosurveillance of hematologic malignancies (23). NK cell effector function is regulated by the balance between inhibitory ligands, mainly canonical and non-canonical MHC-I, and activating ligands presented by the target cell at the IS. Down-modulation of MHC-I is a common feature on cancerous cells and, if accompanied by upregulation of stress-induced activating ligands, leads to activation of NK cells and subsequent target cell lysis (24). Sufficient activating signal results in the release of cytokines, such as IFN-γ and TNF-α, and the formation of a lytic IS that includes directed degranulation of cytotoxic molecules, such as perforin and granzyme B, towards the conjugated target cell (23). CLL immune evasion from NK cells has been described to occur mainly through the upregulation of non-canonical MHC-I isoforms HLA-G and HLA-E (39–41). The NK cell repertoire of an individual can be defined by the simultaneous expression of different receptors and is quite diverse with up to 30’000 different phenotypic populations. Interestingly, the expression of the HLA-G receptor KIR2DL4 is universally found on all NK cells  (40, 42, 43). This indicates that overexpression of HLA-G on CLL cells can provide immune evasion from any NK cell subpopulation. Accordingly, monoclonal antibody blockade therapies targeting HLA-E or HLA-G overexpression successfully increased the natural cytotoxicity of NK cells from CLL patients *in vitro* (40, 44). However, commercial HLA-E monoclonal antibodies are not specific and show cross-reactivity with HLA-A/B/C (45) and HLA-G is characterized by the presence of several isoforms and a high intra- and interpatient heterogeneity, making it a difficult target (23). Alternative inhibition of the inhibitory HLA-E receptor NKG2A showed promising results *in vitro* (46) and is currently tested in several clinical trials, however a phase I/II study of Monalizumab in combination with Ibrutinib including CLL patients was terminated in 2018 (NCT02557516).

Even though NK cell expansion in CLL patients has been reported, these NK cells are described to be hyporesponsive due to a downregulation of activating receptors. They also show a reduced degranulation efficiency against malignant B lymphocytes, through both natural or antibody dependent cell cytotoxicity (ADCC) triggered by rituximab (23). The exhausted NK cell phenotype is enhanced in patients with a progressive disease and results in a loss of NK cell cytotoxicity against CLL target cells. However, CLL patients’ exhausted NK cells can be replaced by activated NK cells coming from a healthy donor. Such allogenic adoptive cell therapy studies showed that unmutated CLL cells are susceptible targets for activated NK cells (46, 47). As demonstrated for other hematologic malignancies, cytotoxic lymphocytes expressing chimeric antigen receptors (CARs) can efficiently lyse tumor cells, and in an attempt to circumvent toxic side effects of CAR-T cell therapy, anti-CD19 CAR-NK cells have been tried for B cell malignancies (48). While this new therapeutic approach holds promising results in first clinical trials (48), little is known about the CAR IS (49). Although some preliminaries studies suggest that CAR IS are superior to conventional NK/T cells IS (50, 51), it remains unclear whether these IS can also be affected by resistant subpopulations of tumors that can modulate IS formation or functions.

These new treatment options set the focus on the lytic IS formed between CLL cells and NK cells and the underlying resistance mechanisms that could result in disease progression. Actin cytoskeleton remodeling has recently emerged as an important process underlying evasion of solid tumor cells, such as breast cancer cells, from NK cell cytotoxicity (23, 24, 52–55). However, the role of the actin cytoskeleton in CLL cells during NK cell attack has not been evaluated so far. Here, we show that a subset of CLL cells from four cell lines, but also patient-derived cells respond to NK cell attack by fast polarization of actin filaments at the IS. Live cell imaging and imaging flow cytometry analyses suggest that synaptic actin accumulation protects CLL cells against NK cell-mediated killing by reducing intracellular levels of granzyme B. Remarkably, pharmacological inhibition of an actin regulatory pathway in CLL cells was sufficient to prevent actin cytoskeleton remodeling, promote granzyme B accumulation, and restore high susceptibility to NK cell-mediated cytotoxicity. Similar results were obtained with patient-derived CLL cells that showed reduced resistance to NK cell-mediated cell death after inhibition of actin dynamics. In this context, blocking antibodies targeting HLA-G also demonstrated that release of the inhibitory interaction of HLA-G with its receptor in NK cells improves conjugate formation. Our data support that interfering with actin cytoskeleton remodeling in CLL cells in combination with antibody blockade provides an opportunity to restore a potent NK cell anti-tumor response in aggressive CLL.

## Methods

### Cell Lines and Cell Culture Conditions

The CLL cell lines used in this study were purchased from DSMZ (German Collection of Microorganisms and Cell Cultures GmbH, Braunschweig, Germany). Cell lines were authenticated through STR profiling analysis (Microsynth, Switzerland) or purchased directly from DSMZ. HG-3, PGA-1, JVM-3 and MEC-1 cell lines were cultured in RPMI-1640 (ThermoFisher Scientific, cat. # 61870010) supplemented with 10% (v/v) fetal bovine serum (FBS, Life Technologies, cat. #10500-064), 100 U/mL penicillin and 0.1 mg/ml streptomycin (Westburg, cat. #LO DE17-602E). The NK-92MI cell line was kept in RPMI-1640 supplemented with 10% (v/v) FBS, 10% (v/v) horse serum (ATCC, cat. # 30-2040), 100U/ml penicillin and 0.1 mg/mL streptomycin. All cell lines were cultured under humidifying conditions at 37°C and 5% CO_2_ and were checked routinely for mycoplasma contamination using the MycoAlert detection kit (Lonza, cat. # LT07-318).

### Isolation of Human Primary NK Cells

Peripheral blood mononuclear cells (PBMCs) were isolated from buffy coats from healthy, anonymous donors provided by the Luxembourg Red Cross. Upon receipt, buffy coats were diluted ten times with Ca^2+^/Mg^2+^ free phosphate buffered saline (PBS) and the low-density PBMC fraction was isolated by centrifugation over a Lymphoprep density gradient (Stemcell Technologies, cat. # 07861). After centrifugation, the PBMC layer was collected, washed several times with Ca^2+^/Mg^2+^ free PBS and red blood cells were lysed with ACK buffer (ThermoFisher Scientific, cat. # A1049201). Following erythrocytes lysis, cells were washed once with Ca^2+^/Mg^2+^ free PBS, counted with Trypan blue and cell concentration adjusted for NK cell isolation. NK cells were isolated with the MojoSort human NK cell isolation kit (BioLegend, cat. # 480054) combined with a LS column (Miltenyi Biotec, cat. # 130-042-401). Isolated NK cells were cultured overnight in RPMI 1640 supplemented with 10% FBS, 10 mM HEPES (ThermoFisher Scientific, cat. # 15630056), 100 U/mL penicillin, 0.1 mg/mL streptomycin, 100 U/mL recombinant human interleukin-2 (IL-2; Peprotech, cat. # 200-02) and 10 ng/mL recombinant human IL-15 (IL-2; Peprotech, cat. # 200-15).

### Isolation of Human Primary CLL Cells

Peripheral blood samples were collected from anonymous CLL patients. All samples used in this study were obtained after informed consent in accordance with the Declaration of Helsinki and the Comité National d’Ethique de Recherche Luxembourg (CNER No. 201707/02 Version 1.2). CLL was diagnosed according to standard clinical criteria. PBMCs were isolated from fresh blood samples using standard density centrifugation over a Lymphoprep gradient. Isolated cells were washed twice in Ca^2+^/Mg^2+^ free PBS and suspended in complete medium (RPMI 1640 supplemented with 10% FBS, 100 U/mL penicillin, and 0.1 mg/mL streptomycin). PBMCs were either used immediately or were cryopreserved in FBS with 10% DMSO. After thawing, cells were allowed to recover overnight before being used for further experiments.

### Cell Transduction and Cdc42 Inhibition

mEmerald-Lifeact-7 was a gift from Dr. M. Davidson (Addgene plasmid # 54148). For generation of stable cell lines, the mEmerald-Lifeact fragment was subcloned into the viral pCDH-EF1α-MCS-IRES-puro plasmid (System Biosciences, cat. # CD532A-2). Infectious particles were produced using HEK293 cells and used to infect HG-3, PGA-1, JVM-3, and MEC-1cell lines. Transduced cells were selected with puromycin (0.5 μg/ml, Sigma-Aldrich, cat. #P8833).

To inhibit Cdc42 activity in CLL cell lines, the cells were incubated for 1 h with 50 µM of the cell-permeable Cdc42 inhibitor ZCL278 (Sigma Aldrich Merck Calbiochem, cat. # 500503)  (56). Cells were washed after treatment and allowed to recover for 1 h or 5 hrs in complete medium before stimulation with human recombinant EGF (0.1µg/mL; PeproTech, cat. # AF-100-15) for 15 min. Inhibition of Cdc42 activity upon stimulation was confirmed using a Cdc42 G-LISA Activation Assay following the manufacturer protocol (Cytoskeleton Inc., cat. # BK127-S).

### Cytotoxicity Assay

For cytotoxicity assays, NK cells (effectors) were counted and stained with anti-human CD56-PE/Cy7 (BioLegend, cat. # 318318, clone HCD56). Effector cells were co-cultured with mEmerald-Lifeact^+^ HG-3, PGA-1, JVM-3 or MEC-1 (target cells) at effector-target (E:T) ratios of 1:1 and 5:1 for 4 hrs at 37°C/5% CO_2_. After incubation, the plate was placed on ice in the dark to stop the experiment until acquisition on the flow cytometer. Immediately before acquisition on a CytoFLEX (Beckman Coulter), TO-PRO-3 Iodide (ThermoFisher Scientific, cat. # T3605) was added to the samples (0.05 μM final concentration). Generated data were analyzed with FlowJo v10.6.2. software.

### Flow Cytometry

To assess cell death in target cells, mEmerald-Lifeact^+^ HG-3, PGA-1, JVM-3 or MEC-1 target cells were incubated for 45 min with CD56-PE/Cy7-labeled NK-92MI cells. Cells were washed with cold Annexin V binding buffer (Biolegend, cat. # 422201) twice. Afterwards, cells were resuspended in 100 μl Annexin V binding buffer with 5 μl Alexa Fluor^®^ 647 Annexin V (Biolegend, cat. #640912) and 5 μl propidium iodide staining solution (Sigma-Aldrich, cat. #P4864) per million cells. Cells were incubated for 15 min at RT in the dark, before addition of 400 μl Annexin V binding buffer and analysis by flow cytometry on a CytoFLEX (Beckman Coulter). Generated data were analyzed with FlowJo v10.6.2. software.

### Imaging Flow Cytometry

For conjugate formation, NK cells were counted and stained with anti-human CD56-PE/Cy7 (BioLegend, cat. # 318318, clone HCD56), before co-culture with mEmerald-Lifeact**^+^** target cells at an E:T ratio of 3:1 in the presence of Hoechst 33342 (0.5 μg/mL final; Miltenyi Biotec, cat. # 130-111-569). Conjugation was allowed for 40 min at 37°C before fixation with 2% paraformaldehyde (PFA; Agar scientific, cat. # R1026) for 15 min at 37°C, and permeabilization with 0.1% Triton X-100 (Sigma Aldrich, cat. # T9284) for 10 min at room temperature (RT). Prior to intracellular staining, samples were washed twice with PBS and then stained for anti-Granzyme B-APC (BioLegend, cat. # 372204, clone QA16A02).

To analyze apoptosis in target cells, cells were centrifuged after 30 min of co-incubation and stained with Zombie Red (BioLegend, cat. # 423110) in PBS for 10 min at RT. Cells were then washed with cold cell staining buffer (BioLegend, cat. # 420201), resuspended in 100 μl Annexin V binding buffer (BioLegend, cat. # 422201) with 5 μl Alexa Fluor^®^ 647 Annexin V (BioLegend, cat. # 640943) and stained for 15 min at RT in the dark. Cells were then washed with Annexin V binding buffer and fixed in 2% v/v PFA diluted in Annexin V binding buffer. After fixation, cells were washed in Annexin V binding buffer and kept at 4°C in this buffer until acquisition. For acquisition, ImageStream^®^X Mark II (EMD Millipore) with four built-in lasers (405 nm, 488 nm, 561 nm, 642 nm) and the INSPIRE^®^ software (EMD Millipore) were used. Analysis for AR, including the gating strategy, masks and features, were described previously (52) and are shown in **Supplementary Figures S1C, D**.

For analysis of patient-derived CLL cells, PBMCs were stained for 30 min with 0.2 µM of the cell permeable F-actin probe SiR-actin (Spirochrome AG, cat. #SC001). To inhibit Cdc42 activity, cells were then treated for 1 h with 50 µM of ZCL278 or vehicle control in complete medium. Before co-culture with NK-92MI cells, patient CLL cells were stained with anti-human CD19-FITC (BioLegend, cat. #302256, clone HIB19), anti-human CD5-BV605 (BioLegend, cat. #364019, clone L17F12), and 10 μg/mL anti-human HLA-G (BioLegend, cat. #335902, clone 87G) or control IgG (BioLegend, cat. #400201, clone MOPC-173) for 30 min at 4°C. Cells were allowed to conjugate with NK-92MI for 30 min in the presence of Hoechst 33342 (0.5 μg/mL final concentration), before staining with 0.1X Live-or-Dye NucFix™ Red for 15 min in PBS. Conjugates were washed in PBS containing Ca^2+^/Mg^2+^ and fixed with 2% PFA for 15 min at 37°C. For analysis of AR and cell death, CLL cells were identified as CD19+/CD5+ cells in conjugation with CD56+ NK-92MI cells.

### Confocal Microscopy

For labelling, mEmerald-Lifeact^+^ target cells were settled on a Poly-L-Lysin (25 µg/mL, Sigma-Aldrich, cat. # P4707) coated µ-slide 8 well (Ibidi, cat. # 80826) for 10 min before fixation with 2% paraformaldehyde (PFA). Cells were permeabilized with 0.1% Triton X-100 and labelled with anti-α-tubulin antibody (Sigma-Aldrich, cat. # T5168, clone B-5-1-2), goat-anti-mouse Alexa Fluor 633 (Invitrogen, cat. # A-21126) and with acti-stain 555 phalloidin (100 nM, Cytoskeleton Inc., cat. # PHDH1) and DAPI (0.2 μg/mL, Sigma-Aldrich). For conjugate formation, NK cells were counted and stained with the CellTracker™ Orange CMRA dye (1 µM, Invitrogen, cat. # C34551), before co-culture with mEmerald-Lifeact target cells at an E:T ratio of 1:1 in the presence of Hoechst 33342 (0.5 μg/mL final). Conjugation was allowed for 40 min at 37°C, then cells were settled on a Poly-L-Lysin coated µ-slide 8 well for 10 min before fixation with 2% PFA. After 2 washings, PBS was replaced by mounting medium (Ibidi, cat. # 50001) before cell imaging. For acquisition, high-resolution pictures were acquired on a Zeiss LSM880 fastAiry confocal microscope, in the Airy mode. A multitrack configuration was used with laser 405 nm, 488 nm, 543 nm, and 633 nm for excitation. A stack of 50 slices with an interval of 0.2 µm was acquired. The fluorescence intensity was measured on the maximum intensity projection picture of the stack in a rectangle of 5 µm width set in the center of the IS, with the macro “GetProfileExample” in the Image J v1.53e software.

For live cell imaging, NK92MI cells were counted and stained with the CellTracker™ Orange CMRA dye (1 µM), before co-culture with mEmerald-Lifeact target cells at an E:T ratio of 1:1 in the presence of SYTOX™ Blue (2 µM, Invitrogen, cat. # S11348). For acquisition, cells were maintained under the microscope at 37°C and 5% CO_2_. A single-track configuration was used with excitation at 405 nm, 488 nm, and 543 nm. The pinhole was open to acquire a 2 µm depth slice. A stack of 4 slices with an interval of 2 µm was acquired for 1 h at a rate of one picture every 4 min.

### Statistical Analysis

The paired Student’s t-test and 2-way ANOVA in Prism 9 (GraphPad) were used to determine the statistical significance of the results obtained. For apoptosis experiments, a Z-score test for two population proportions was used to determine the statistical significance between samples. * p ≤ 0.05, ** p ≤ 0.01 and *** p ≤ 0.001.

## Results

### CLL Cell Resistance to NK Cell-Mediated Cytotoxicity Correlates With Actin Cytoskeleton Polarization to the Immunological Synapse

**Figure 1:**
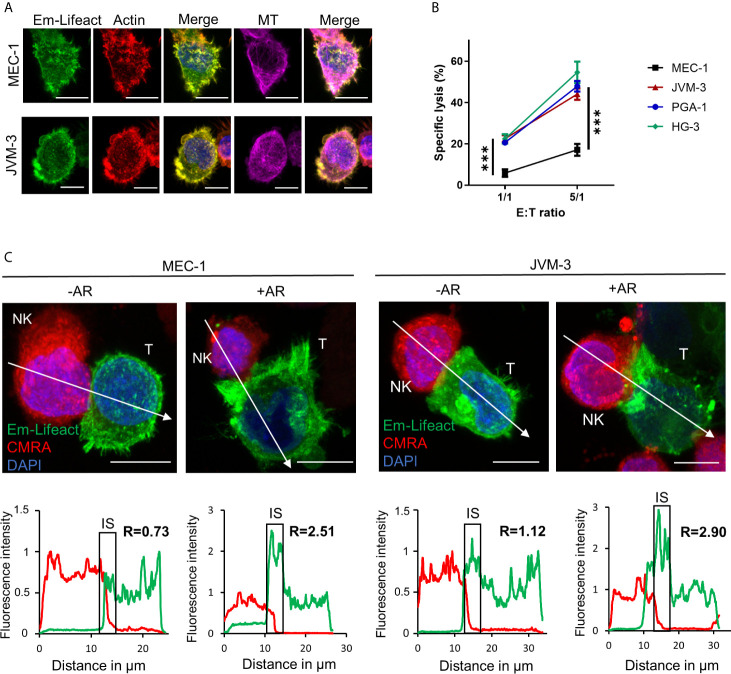
CLL cells have the ability to respond to NK cell attack with an actin response associated to their resistance. **(A)** JVM-3 and MEC-1 cells were transduced to express the actin cytoskeleton marker Emerald-Lifeact (green). Stable cell lines were stained with Acti-stain 555 phalloidin (red) and anti-tubulin antibody (MT, violet). The yellow-green signal shows the co-localization of the two actin cytoskeleton probes. Bars: 10 µm. **(B)** Cytotoxicity assays with four CLL target cell lines and effector NK-92MI cells at 1:1 and 5:1 E:T ratios for 4 hrs. 2-way ANOVA was applied to determine statistical significance; *** denotes p < 0.0001. **(C)** Confocal microscopy pictures of MEC-1 (left) and JVM-3 (right) cells (T) in conjugation with NK-92MI cells (NK) with and without an actin response. The charts below show the relative fluorescent intensity of Emerald-Lifeact and CMRA along the trajectories (white arrow). The fluorescence was normalized to 1 at the opposite site of the synapse. The region of the immunological synapse is indicated with “IS”. Compared to the opposing end, target cells with an actin response have a more than 2-fold higher fluorescent signal at the IS. Bars: 10µm.

To evaluate actin cytoskeleton organization and dynamics in aggressive forms of CLL, three IGHV mutated cell lines, namely PGA-1, JVM-3 and MEC-1, and one IGHV non-mutated cell line (HG-3) (**Supplemental Figure S1A**) were modified to stably express the mEmerald-tagged actin marker Lifeact (**Figure 1A**) (57). With this approach, labeling of the actin cytoskeleton became obsolete and a spill-over from the NK cell actin cytoskeleton could be avoided. Cytotoxicity assay found MEC-1 as a highly resistant CLL cell line compared to the other three CLL cell lines (**Figure 1B**). After 4 hrs of co-culture with an excess of NK-92MI cells at a 5:1 E:T ratio, MEC-1 cells were lysed with an average rate of 17%. In comparison, HG-3, PGA-1, and JVM-3 cells were significantly more susceptible with average NK cell-specific lysis rates of 54%, 47%, or 44%, respectively. Using confocal microscopy, we found that some NK cell-conjugated CLL cells showed a strong polarization of filamentous actin to the synaptic area (**Figure 1C** and **Supplementary Figure S1B**). We recently reported similar synaptic accumulation of filamentous actin during NK cell attack in breast cancer cells and termed this phenomenon “actin response” (AR) (52). Analysis of confocal microscopy images revealed that CLL cells with an AR exhibit a more than 2-fold increase of F-actin at the IS as compared to CLL cells without an AR (**Figure 1C** and **Supplemental Figure S1B**). CLL cells without an AR showed a relatively homogenous distribution of F-actin.

**Figure 2:**
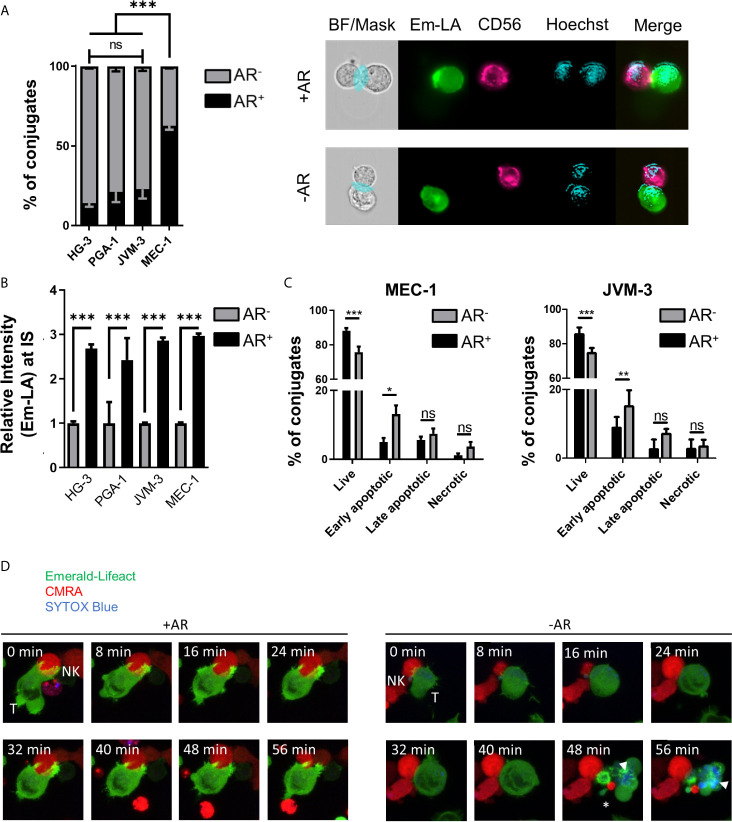
Quantification and functional consequence of the actin response during NK cell attack. **(A)** Quantitative Imagestream analysis of CLL-NK cell conjugates. CLL cells HG-3, PGA-1, JVM-3 and MEC-1 were analyzed for their actin response frequency in conjugates with NK-92MI cells. Percentages of target cells in conjugation with NK-92MI cells with (black, AR^+^) and without (grey, AR^-^) an actin response. *** denotes p < 0.0001 **(B)** Relative intensity of Emerald-Lifeact at the IS in target cells conjugates with NK cells with respect to absence (grey, AR^-^) to presence (black, AR^+^). Data represents results of 6 different experiments and plots over 2000 conjugates per cell line. Data was normalized to conjugates without an AR. *** denotes p < 0.0001 **(C)** Imagestream analysis of target cell death in CLL target cells conjugated with NK cells in the presence (black, AR^+^) or absence (grey, AR^-^) of an actin response. Target cell death was assessed by Annexin V and propidium iodide staining. * denotes p<0.05, ** denotes p< 0.001, *** denotes p < 0.0001 **(D)** Time lapse imaging of actin dynamics in MEC-1 CLL cells upon NK cell attack. The AR^+^ target cell can resist NK cell-induced cell death. Target cells not capable to produce an actin response are effectively lysed as seen by the SYTOX blue staining (white arrow head) and disappearance of normal cellular structures and membrane blebbing (asterisk). ns, non significant.

Quantitative analysis of the relative number of NK cell-conjugated CLL cells with and without an AR was conducted using high-throughput imaging flow cytometry. For analysis of conjugates, 5x10^3^ double-positive events were acquired per experiment with the same settings. After quality control, over 1000 conjugates between CLL and NK cells from 3 independent experiments were evaluated for the presence or absence of an AR (**Supplementary Figures S1C, D**). Our data revealed that a majority (about 63%) of highly resistant MEC-1 cells exhibited an AR, while in more susceptible CLL cell lines only a small fraction exhibited this phenotype (**Figure 2A**). HG-3 cells showed the lowest rate of AR with an average of only 14% of conjugated CLL cells forming an AR, while PGA-1 and JVM-3 had an AR frequency of 21 and 23%, respectively. To better characterize and compare the AR in CLL cells, we analyzed both the total F-actin content in AR^-^ and AR^+^ cells, as well as the relative signal intensity of Emerald-Lifeact within the synaptic region of each type of cells. The results show that the total F-actin content is not significantly different in conjugated CLL cells with or without an AR (**Supplementary Figure S2A**). However, in CLL cells with an AR, the relative intensity of F-actin at the IS was increased almost 3-fold (**Figure 2B**), indicating a prominent polarization of the actin cytoskeleton toward NK cells. Additionally, these data suggest the accumulation of F-actin at the IS is compensated by equivalent depolymerization of actin filaments in other parts of the cells, resulting in no net increase of the overall F-actin content in target cells.

To further characterize the link between the AR and resistance to NK cell-mediated death, individual cell-cell conjugates were analyzed using imaging flow cytometry after tumor cells were labeled with the live/dead cell discrimination marker Zombie Red and Annexin V. Early apoptotic cells were characterized as Annexin V^+^/Zombie Red^-^, late apoptotic cells as Annexin V^+^/Zombie Red^+^, and Annexin V^-^/Zombie Red^+^ cells were classified as necrotic for quantitative analysis. In both MEC-1 and JVM-3 cell lines, AR^+^ cells showed significantly less signs of apoptosis, especially early apoptosis, than AR^-^ cells (**Figure 2C**). Similar results were obtained for the other two CLL cell lines HG-3 and PGA-1 (**Supplementary Figure S2B**). Since NK and target cells were allowed to conjugate for only 45 minutes, induction of primarily early apoptosis is within the time frame of normal NK cell cytotoxic activity (58, 59). The protective effect of the AR was similar in all cell lines. Yet, it is important to consider that the size of the AR^+^ cell subpopulation greatly differs between the MEC-1 cell line and the HG-3, PGA-1, and JVM-3 cell lines, explaining the difference in their overall susceptibility.

Live cell imaging analysis revealed that the AR in CLL cells is induced immediately after their first physical contact with NK cells and persisted throughout the whole cell-to-cell interaction time (**Figure 2D** left and **Supplementary Movie 1**). In addition, it provides direct evidence that NK cell-conjugated CLL cells that successfully assembled an AR survived the immune cell attack, while those that failed to mount an AR were efficiently lysed, as shown by uptake of SYTOX blue viability dye (**Figure 2D** right, **Supplementary Movie 2** and **Supplementary Figure S2C**).

In conclusion, we identified a subpopulation of cells in four CLL cell lines that responds to NK cell attack with fast polarization of the actin cytoskeleton to the IS (or AR), a process that closely correlates with resistance to NK cell-mediated lysis. Thus, the overall susceptibility of a given cell line can be directly deduced from the relative size of this subpopulation within the cell, with a large subpopulation being predictive of a highly resistant phenotype.

### Targeted Inhibition of Actin Remodeling in CLL Cells Restores High Susceptibility to NK Cell-Mediated Killing

The Rho GTPase cell division control protein 42 homolog (CDC42) is a key regulator of actin polymerization and cell polarity (60). It promotes F-actin polymerization in association with the neuronal Wiskott-Aldrich syndrome protein (N-WASP) and the Arp2/3 complex (61). In addition, CDC42 localizes the N-WASp-Arp2/3 complex close to the cell membrane through interaction with phosphatidylinositol (4, 5) bisphosphate (62). In an attempt to inhibit fast actin remodeling in CLL cells during NK cell attack and to confirm the causal relation between the AR and CLL cell-intrinsic resistance to NK cell-mediated lysis, CDC42 was pharmacologically inhibited using the cell-permeable CDC42-specific inhibitor ZCL278 (56). 50 µM of the inhibitor was found to achieve significant and sustained inhibition of CDC42 activity, without inducing significant toxicity (**Supplementary Figure S2D**). To avoid side effects on the actin cytoskeleton of NK cells, CLL cells were pre-treated with ZCL278 and the drug was washed out before co-culture with NK cells for following experimental assays (63).

**Figure 3:**
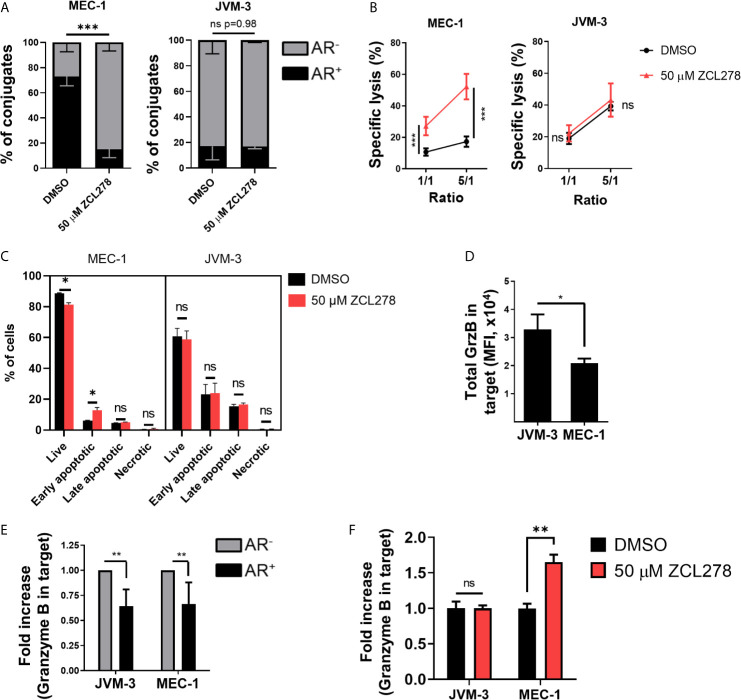
Pharmacological inhibition of Cdc42 increases CLL cell susceptibility to NK cell attack by lowering actin response frequency. **(A)** Quantitative Imagestream analysis of CLL-NK cell conjugates. CLL cells were pre-treated with 50 µM ZCL278 and analyzed for their actin response frequency in conjugates with NK-92MI cells. *** denotes p < 0.0001 **(B)** NK cell-mediated cytotoxicity against DMSO- or ZCL278-treated CLL cells. Pre-treated JVM-3 and MEC-1 cells were co-cultured for 4 hrs with NK-92MI cells at E:T ratios of 1:1 and 5:1. Cell death was evaluated by To-Pro-3 staining and adjusted to NK cell-specific lysis. *** denotes p < 0.0001 **(C)** Flow cytometry analysis of DMSO- or ZCL278-pretreated CLL target cells after 45 minutes of co-culture with effector NK-92MI cells at a 1:1 E:T ratio. Apoptosis was evaluated by Annexin V and PI staining. * denotes p < 0.05, ** denotes p < 0.001, *** denotes p < 0.0001 **(D)** Imagestream analysis of total granzyme B load in target cells conjugated to NK-92MI cells after 45 minutes of co-culture. * denotes p < 0.05 **(E)** Target cells were categorized into AR^+^ (black) and AR^-^ (grey) and granzyme B load in target cells evaluated. Data was normalized to AR^-^ conjugates. ** denotes p < 0.001 **(F)** Imagestream analysis of intracellular granzyme B in target cells after ZCL278-induced Cdc42 inhibition. ** denotes p < 0.001. ns, non significant.

Inhibition of CDC42 activity in the resistant MEC-1 cell line resulted in potently impaired AR formation with a more than four-fold decrease in the relative number of conjugated cells exhibiting an AR as compared to DMSO-treated control cells (16.9% and 73.3%, respectively) (**Figure 3A**, left). Remarkably, such an effect was paralleled by an almost five-fold increase in tumor cell susceptibility to NK cell-mediated lysis. Indeed, 52.2% of ZCL278-treated MEC-1 cells were lysed at a 5:1 E:T ratio, while only 10.6% of DMSO-treated cells were lysed in the same conditions (**Figure 3B**, left). Thus, inhibition of *de novo* F-actin polymerization and the AR in CLL cells was sufficient to turn the initially highly resistant MEC-1 cell line into a highly susceptible phenotype. Spontaneous cell death did not change in response to ZCL278 treatment (**Supplementary Figure S2E**), indicating indeed increased susceptibility of MEC-1 cells to NK cell-mediated lysis after CDC42 inhibition. Pharmacological inhibition of CDC42 in HG-3, PGA-1, or JVM-3 cells could not further reduce the small subpopulation of CLL cells with an AR in these cell lines (**Figure 3A**, right and **Supplementary Figure S3A**) and had accordingly no effect on their already highly susceptible phenotype (**Figure 3B**, right and **Supplementary Figure S3B**).

Then MEC-1 cells were pre-treated with DMSO or 50 µM ZCL278 prior to 45 min co-culture with NK cells (1:1 E:T ratio), subsequent labelling with AnnexinV and propidium iodide and quantification of target cell killing by standard flow cytometry. Inhibition of the AR using ZCL278 resulted in a significant increase in apoptotic MEC-1 cells, especially early apoptotic cells, compared to DMSO-treated control cells (**Figure 3C**). The percentage of early apoptotic cells increased from 5.8% to 15%, a value that parallels the imaging flow cytometry analysis of cell death in AR^-^ MEC-1 conjugates with NK cells (**Figure 2C**), indicating restoration of a susceptible phenotype. Longer incubation time points did not change the distribution of the populations significantly, as late apoptotic and necrotic cells were removed during the washing steps (data not shown). Consistent with our previous results and the intrinsically low AR frequency in the other CLL cell lines, ZCL278 treatment did not significantly modify apoptosis in these cell lines (**Figure 3C** and **Supplementary Figure S3C**). Altogether, these results indicate that the AR is mediated by CDC42 dependent actin polymerization and that inhibition of CDC42 activity potently restores CLL cell susceptibility to NK cell-mediated cytotoxicity.

### Synaptic Actin Remodeling Leads to Reduced Granzyme B Levels in NK Cell-Conjugated CLL Cells

Direct cytotoxicity of NK cells occurs through the release of cytotoxic granules (23, 24). These granules contain among others granzymes and perforin that trigger cancer cell lysis through formation of membrane lesions and induction of caspase-3 and caspase-8 activation. We assessed the levels of one key granzyme, namely granzyme B, transferred into CLL cells. On average, JVM-3 cells in conjugation with NK cells showed a higher intracellular intensity of granzyme B compared to MEC-1 cells (**Figure 3D**), which is consistent with the respective cell line susceptibility to NK cell-mediated lysis and ability to remodel actin cytoskeleton following immune attack. Moreover, in all four cell lines, intracellular levels of granzyme B were considerably reduced (by approximately 35%) in the cell subpopulation exhibiting an AR as compared to the cell subpopulation without an AR, suggesting that the AR leads to reduced amounts of granzyme B transferred to target cells (**Figure 3E** and **Supplementary Figure S3D**).

To assess if inhibiting the AR could restore elevated levels of granzyme B in MEC-1 cells, MEC-1 cells were pre-treated with 50 µM ZCL278 to lower the cell subpopulation with an AR and granzyme B levels were quantified after 45 minutes incubation with effector cells (**Figure 3F** and **Supplementary Figure S3E**). The results show that AR inhibition increased the intracellular granzyme B intensity to levels comparable to JVM-3 cells. Altogether our data provide strong indication that the AR protects CLL cells from cytotoxicity mediated by NK cells through the granzyme B/perforin pathway.

### Primary NK Cells Induce the Actin Response and Confirm CLL Cell Line Intrinsic Actin Response Frequency

The NK-92MI cell line used in this study is a CD16^-^ effector cell lines that additionally lacks major inhibitory receptors such as Killer-cell immunoglobulin-like receptors (KIR) and NKG2A receptors (64, 65). These results in induction of natural cytotoxicity through recognition of activating ligands such as MHC class I homologues MIC-A (MIC-A), MIC-B, and UL-16 binding protein (ULBP) on target cells and interaction of lymphocyte function-associated antigen 1 (LFA-1) with its ligand intercellular adhesion molecule 1 (ICAM-1). According to its “hyperactive” phenotype, the NK cell line kills target cells in an unrestricted manner and with low specificity. Thus, we re-evaluated the AR in CLL cell lines challenged with primary NK cells isolated from healthy donors.

**Figure 4:**
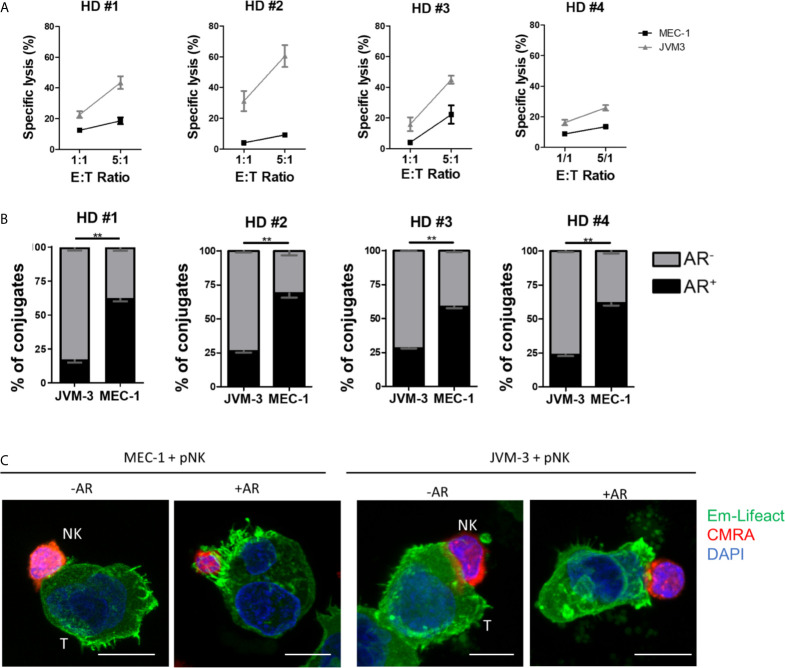
Primary human NK cells can invoke the actin response in MEC-1 and JVM-3 cells. **(A)** Cytotoxicity assay with primary NK cells isolated from four healthy human donors and JVM-3 and MEC-1 CLL target cells at 1:1 and 5:1 E:T ratios. Target cells and primary NK cells were co-cultured for 4 hrs before analysis of NK cell-specific lysis by flow cytometry. **(B)** Quantitative analysis of primary NK-CLL conjugates with (black, AR^+^) and without (grey, AR^-^) actin response by imaging flow cytometry. For each donors a minimum of 200 conjugates were analyzed. ** denotes p < 0.001 **(C)** Representative confocal microscopy pictures of primary NK cells (CMRA, red) in conjugation with MEC-1 (left) and JVM-3 (right) cells with or without an actin response. Bars: 10 µm.

To this end, primary NK cells were isolated from PBMCs using a negative selection kit reaching a purity of >90% (**Supplementary Figure S3F**) and kept in culture overnight with IL-2 and IL-15 for activation. Cytotoxicity of primary NK cells against JVM-3 and MEC-1 target cells was lower compared to lysis rates achieved with the NK-92MI cell line. However, the previously established difference in intrinsic susceptibility between the two target cell lines was confirmed (**Figure 4A**). Indeed, despite inter-donor variability, JVM-3 cells were more effectively lysed by donor-derived NK cells than MEC-1 target cells.

Quantitative analysis of the AR frequency with primary NK cells using imaging flow cytometry resulted in remarkably comparable results as seen with the NK-92MI cell line (**Figure 4B**), with about 25% of AR^+^ conjugates with JVM-3 cells and 60-71% of conjugated MEC-1 cells showing an AR. Confocal microscopy provided direct evidence that primary NK cell attacks also invoked an AR in some individual CLL cells (**Figure 4C**).

In conclusion, activated donor-derived healthy NK cells induce an AR in CLL cells at a same frequency as the NK-92MI cell line, supporting that the AR is a process intrinsic to the CLL cells and is not dependent on the origin of the effector NK cells, being a cell line or isolated form a healthy donor.

### Inhibition of the Actin Response in Combination With HLA-G Blocking Antibody Restores Patient-Derived CLL Cell Susceptibility to NK Cell-Mediated Killing

**Figure 5:**
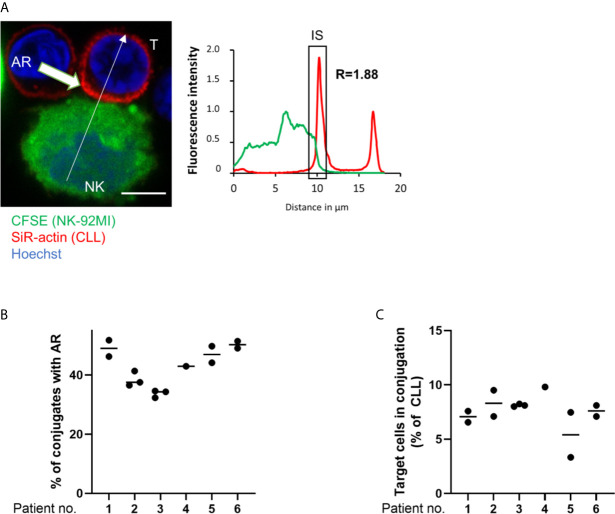
The actin response in patient-derived primary CLL cells. **(A)** Representative confocal microscopy image of an immune synapse between a primary CLL cell (T) and NK-92MI cell (NK). The chart to the right shows the relative fluorescent intensity of SiR-actin along the trajectory (white arrow). The fluorescence was normalized to 1 at the opposite site of the synapse. The region of the immunological synapse is indicated with “IS”. Bar: 10 µm **(B)** Quantitative imaging flow cytometry analysis of the AR in primary CLL cells in conjugation with NK-92MI effector cells. CLL cells were identified as CD5^+^/CD19^+^ cells. Actin staining was performed using spirochrome labelling. **(C)** Quantification of primary CLL cells in conjugation with NK-92MI cells after 45 minutes of co-culture as percentage of total target population.

To investigate if primary CLL cells mount an AR and if inhibition of the latter improves their susceptibility to NK cell-mediated cytotoxicity, CLL patient samples (n=10) were analyzed in a series of *ex vivo* assays. In these assays, PBMCs were isolated from peripheral blood and their actin cytoskeleton was stained with SiR-actin, a cell-permeable and F-actin specific probe. As illustrated in **Figure 5A**, ARs were observed in primary CLL cells conjugated with NK-92MI cells. These ARs were of slightly lower intensity compared to those seen with CLL cell lines, with a roughly 1.8-fold increase of fluorescence intensity for F-actin at the IS as compared to the opposing cell side. This could be explained by the smaller cell size of primary CLL cells. The AR was then quantified in CLL cells originating from six patients using imaging flow cytometry. Our results revealed a similar and relatively high rate of AR in all primary CLL cell samples with values ranging from 38.4% to 51.2% of analyzed primary CLL-NK-92MI cell conjugates (**Figure 5B**). We noticed that the conjugation rate of primary CLL cells with NK-92MI cells was particularly low (~7% of all CD5^+^/CD19^+^ cells; **Figure 5C**), which can be explained by a high surface expression of HLA-G on *ex vivo* CLL cells (40) and expression of the cognate inhibitory receptor immunoglobulin-like transcript 2 (ILT-2, LILRB1) on NK-92MI cells (66). Interaction of ILT-2 with HLA-G has been reported to negatively impact not only NK cell polarization, but also to interfere with F-actin assembly at the NK cell side of the IS (67) and can thereby prevent conjugate formation between patient-derived CLL and NK-92MI cells (24).

**Figure 6:**
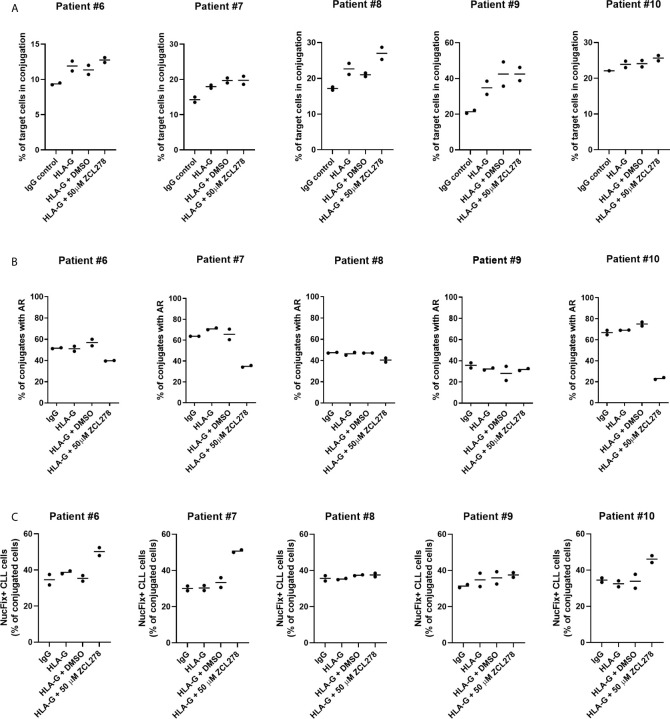
Inhibition of the actin response in combination with anti-HLA-G blocking antibody substantially improves NK cell-mediated killing of CLL cells. **(A)** Conjugation frequency of primary CLL cells as quantified by imaging flow cytometry in the absence or presence of HLA-G blocking antibody. Target cells were incubated with 10 µg/mL control IgG or blocking antibody against HLA-G, and were used either untreated (IgG, HLA-G), DMSO treated, or after incubation with 50 µM ZCL278 for 1 h before conjugation. Effector and target cells were co-cultured at a 3:1 E:T ratio for 45 min at 37°C and fixed with 2 v/v% PFA. Target cells in conjugation are shown as % of total target cells. **(B)** Quantitative analysis of primary CLL-NK cell conjugates with an actin response in the presence of control IgG or anti-HLA-G blocking antibody by imaging flow cytometry. Target cell were either untreated (IgG, HLA-G) or conjugated after treatment with vehicle (DMSO) or 50 µM ZCL278. For each patient, a minimum of 100 conjugates were analyzed. **(C)** Average percentage of Live-or-Dye NucFix™ Red positive primary CLL cells in conjugation with NK-92MI cells in the presence of control IgG or anti-HLA-G blocking antibody. Target cells are either untreated, treated with vehicle control or 50 µM ZCL278.

To improve conjugate formation, primary CLL cells were treated with an anti-HLA-G blocking antibody for 1 h prior to co-culture with NK-92MI cells. It is noteworthy that NK-92MI cells do not express the Fc receptor CD16 (64), excluding the risk of ADCC. As anticipated, HLA-G blocking antibody increased the frequency of target CLL cells in conjugation with NK-92MI cells compared to control IgG, indicating a release of the inhibitory ILT-2 signaling and restoration of IS formation (**Figure 6A**). Additionally, we aimed at evaluating the effect of ZCL278 on primary CLL cells. Inhibition of CDC42 activity with 50 µM ZCL278 in combination with blocking antibody was found to only minimally or not to alter the rate of conjugation in comparison to vehicle control (DMSO). Conversely, treatment with anti-HLA-G antibody did not alter the rate of AR in primary CLL cells, while ZCL278 treatment reduced the AR in three out of five patients (**Figure 6B**). Although this remains speculative, the lack of response in CLL cells originating from patient 8 and 9 could be explained by an initially low AR frequency (patient no. 9) or upregulation of alternative, CDC42-independent actin polymerization pathways (patient no. 8).

Finally, apoptosis in primary CLL cells in conjugation with NK-92MI was evaluated with regards to anti-HLA-G antibody treatment and CDC42 inhibition. Samples treated with 50 µM ZCL278 that previously showed no changes in the AR frequency (**Figure 6B**) demonstrated similar levels of apoptosis as compared to IgG control, HLA-G, or HLA-G in combination with vehicle treated samples from the same patient (**Figure 6C**, patient no. 8 and 9). In contrast, with CLL samples that showed a reduction of the AR in response to CDC42 inhibition (**Figure 6B**), an increased percentage of conjugated primary CLL cells showed signs of apoptosis (**Figure 6C**, patient no. 6, 7, and 10). These results extend our analysis with CLL cell lines and indicate that the AR is a frequent process in primary CLL cells (with more than 50% of NK cell-conjugated CLL cells showing an AR for most patients) and that targeting of this process can substantially increase CLL cell susceptibility to NK cell-mediated cell death.

In conclusion, we report here for the first time AR in patient-derived cancer cells and show that specific targeting of key actin regulators in combination with anti-HLA-G blocking antibody, increases conjugate formation and target cell susceptibility to NK cell-mediated cytotoxicity opening up the possibility of combinational targeting for CLL patients.

## Discussion

Despite substantial recent advances in the therapy of CLL, treatment options, especially for patients diagnosed with an aggressive disease, particularly with *TP53* deletion and/or mutation, are limited. Effectors of both, the adaptive and the innate immunity immune systems, show severe signs of dysfunction that allow for successful immune evasion of malignant B cells. This includes inhibition of cytotoxic CD8^+^ and activated CD4^+^ T lymphocytes and induction of the immune suppressive M2-like monocyte phenotype instead of pro-inflammatory immune sub-populations. Additionally, CLL induces expansion of regulatory T cells (T_Reg_), overall resulting in the development of a tolerogenic environment and disease progression (11, 68). While special attention has been paid investigating the interaction between CLL and T lymphocytes, recent studies focused on NK cells as an alternative target of chemo-immunotherapy. NK cells derived from CLL patients were described as hyporesponsive due to a loss of the mature, CD56^dim^ NK cell population, possibly due to activation-induced apoptosis as a result of constant exposure to malignant B cells. This was accompanied by a downregulation of activating receptors such as NKG2D that affects the natural cytotoxicity of NK cells (23). However, upon sufficient activating signal through CD16, NK cell function can still be induced, showing that CLL-derived NK cells of the CMV-associated NKG2C^+^/CD16^+^ phenotype are fully functional (69). Total NK cell numbers have repeatedly been reported to be elevated in CLL patients compared to healthy controls, often with an emphasis on CMV-related NKG2C^+^/CD56^dim^/CD16^+^ NK cells (23, 70, 71). These phenotypes accordingly cannot explain the lack of anti-tumor response or disease progression in CLL.

The NK cell line we used in the present study is negative for CD16 and a common model of natural cytotoxicity of NK cells. Although these NK cells are fully activated and effectively recognize their targets, a subpopulation of CLL cells was still resistant to NK cell-mediated cytotoxicity. This indicates an additional intrinsic resistance of CLL that allows escape from NK cells that can be activated either through activating ligands or possibly even through ADCC. Here we show that the actin cytoskeleton of CLL cells plays a critical role in the intrinsic capacity of these cells to avoid destruction by degranulating immune effector cells.

The fast synaptic actin remodeling we observed in CLL cells attacked by NK cells strongly resembled the “actin response” or AR previously described for breast cancer cells and was strongly associated with resistance to NK cell cytotoxicity. Independent of IGHV mutational status, CLL cell lines showed a resistant subpopulation that was characterized by the AR. While HG-3, PGA-1, and JVM-3 showed similar results in all experimental assays with a high susceptibility to NK cell-mediated lysis and high intracellular granzyme B load, MEC-1 cells demonstrated a high resistance to NK cell-mediated cytotoxicity and decreased uptake of NK cell-derived granzyme B. We attributed these differences to the relative size of the AR^+^ subpopulation as we were able to show that *de novo* F-actin polymerization on the cancer side of the IS is strongly associated with survival and resistance during NK cell attack. Pharmacological inhibition of CDC42 activity drastically reduced the size of the AR^+^ subpopulation and resulted in an increase of early apoptotic cells and overall cell death in MEC-1 that can be explained by increased amounts of granzyme B transferred into target cells. Although CDC42 is a central actin cytoskeleton regulator, other pathways might be involved in the process of the AR, as suggested by the remaining AR^+^ subpopulation in HG-3, PGA-1, JVM-3 and MEC-1 cells that resisted treatment with the CDC42-specific inhibitor ZCL278 (56).

An interesting aspect that could be worth further investigation is the potential role of *TP53* in enabling the AR, as the four cell lines differ in their *TP53* mutational status. HG-3, PGA-1, and JVM-3 cells have all been reported to express wildtype *TP53*, while MEC-1 are identified as a *TP53^del/mut^* cell line, expressing a truncated 40kDa version of the p53 protein without transcriptional activity. In CLL patients, mutations of *TP53* or loss of one *TP53* allele are associated with a significant decrease in survival and are predictive for an impaired response to chemo-immunotherapy (72). Additionally *in vitro* experiments attributed expression of wildtype *TP53* or mutational *TP53* and/or loss of *TP53* to differential drug response in several CLL cell lines, including JVM-3 and MEC-1 (73–75). Other studies have shown, that rescue of mutational *TP53* function can restore granzyme B-mediated apoptosis in breast cancer through down-modulation of anti-apoptotic proteins (76). However, in these studies, *TP53* reactivation was achieved in cell lines expressing a missense mutational p53, while in many CLL cases with aberrant *TP53* expression, deletion of the short arm of chromosome 17 (del17p13) results in a complete loss of *TP53*, often in association with *TP53* mutations on the other allele (77). Whether *TP53* status is therefore a critical determinant of the AR frequency in CLL will need to be determined with a larger, better defined patient cohort in the future.

Most importantly, we were able to show that the AR is not an artefact of cell lines but can indeed be found in patient-derived CLL cells. In this context, the inhibitory interaction of surface molecules, such as the non-classical MHC-I isoforms HLA-E and HLA-G, with their corresponding ligands on NK cells might have been underestimated in their significance. Even the NK-92MI cell, known to not express key inhibitory receptors such as KIR or NKG2A, showed a deficiency in conjugate formation with patient-derived CLL cells. This dysfunction could be rescued by antibody blockade targeting HLA-G. However, antibody opsonization of target CLL cells had no impact on the frequency of the AR in primary CLL cells. In combination with CDC42 inhibition, anti-HLA-G blocking antibody greatly increased NK cell-induced apoptosis in patient-derived CLL cells. We hypothesize that this is a consequence of the relative size of the AR^+^ population that could be decreased by pharmacological CDC42 inhibition. This is further supported by the observation that in individual patient samples in which this treatment failed to reduce the size of the AR^+^ population, the frequency of CLL cells showing signs of apoptosis was unchanged compared to untreated and DMSO-treated samples. Since NK-92MI cells are CD16^-^, antibody blockade did not trigger ADCC that could explain the increase in target cell death. It is however worth speculating that NK cells capable of ADCC could be even more effective in inducing apoptosis in CLL cells that underwent dual blocking antibody and CDC42 inhibition therapy.

Yet, actin cytoskeleton targeting drugs, such as cytochalasins or latrunculins, show intolerable toxicity with particularly severe adverse effects on cardiac structure and function and are therefore unfit for clinical trials. Experimental drugs that target cancer-specific F-actin components and confirmation of their efficiency *in vivo* (78, 79) demonstrate however, that targeting of the actin cytoskeleton dynamics is a possibility in our search for new innovative cancer drugs. Importantly, targeting of intrinsic immune escape mechanisms such as the AR can only be effective in combination with other therapies, such as immune checkpoint inhibitor blockade and/or opsonization with tumor-targeting antibodies. Without these therapies, cytotoxic lymphocyte activation, but also IS formation that is fundamental to a functional anti-tumor immune response cannot take place.

Overall, NK cells are emerging as a valuable tool for the control of CLL disease progression and reactivation of their cytotoxic capabilities against cancer cells could potentially improve overall outcome. Selective targeting of intrinsic immune escape mechanisms, such as the here described AR, could provide a new line of therapy for the difficult to treat or relapsing CLL subtypes. It is important to highlight that irrespective of the expression status of poor prognostic markers, such as *TP53* and *IGHV*, all four CLL cell lines, as well as all ten patient-derived CLL samples demonstrated an AR^+^ subpopulation that proved to be resistant against pharmacologic inhibition of CDC42 activity. This shows the presence of another signaling pathway allowing cancer cells to maintain resistance against NK cell cytotoxicity. Further studies employing patient cohorts will be needed to address and confirm the clinical importance of the AR in CLL and its therapeutic value. Further it needs to be evaluated whether the hyporesponsive phenotypical state of CLL-patient derived NK cells is revertible or if allogeneic NK cell therapy could benefit from selective targeting of the AR (47), possibly in combination with other immunomodulatory drugs (44, 80, 81).

## Data Availability Statement

The original contributions presented in the study are included in the article/**Supplementary Material**. Further inquiries can be directed to the corresponding author.

## Ethics Statement

The studies involving human participants were reviewed and approved by Comité National d’Ethique de Recherche Luxembourg (CNER No. 201707/02 Version 1.2). The patients/participants provided their written informed consent to participate in this study.

## Author Contributions

HW designed and performed most of the experimental work, analyzed results, and wrote the manuscript. LF, CH, MK, and AB performed experiments and analyzed results. JM performed NK cell isolation. HW, LF, and AL performed patient CLL cell isolation. Patient samples were provided by SD, JF, and GB. JP and EM organized CLL sample collection and contributed to supervise the study. CT supervised the study and wrote the final version of the manuscript. All authors contributed to the article and approved the submitted version.

## Funding support

Work in CT’s group is supported by grants from the National Research Fund (FNR, Luxembourg, ACTIVASION, C19/BM/13579644), La Fondation Cancer (Luxembourg, ACTIMMUNE, FC/2019/02) and Think Pink Lux (Luxembourg). HW is recipient of a PhD fellowship from the National Research Fund (FNR, Luxembourg), Luxembourg (PRIDE15/10675146/CANBIO). AB and MK are recipient of PhD fellowships from Fonds National de la Recherche Scientifique (FNRS, Belgium, Televie #7.4536.19 ACTIPHAGY and Televie #7.4531.20 TRANSACTINg, respectively). AL is supported by a post-doctoral fellowship from the FNRS (Televie #7.4503.19). JP is supported by the National Research Fund (FNR), Luxembourg (INTER/DFG/16/11509946).

## Conflict of Interest

The authors declare that the research was conducted in the absence of any commercial or financial relationships that could be construed as a potential conflict of interest.

## Supplementary Material

Supplementary Figure 1
**(A)** List of CLL cell line characteristics. **(B)** Confocal microscopy pictures of HG-3 and PGA-1 Emerald-Lifeact cells (T) in conjugation with NK-92MI cells (NK) with and without an actin response. Charts below show the relative fluorescent intensity of Emerald-Lifeact and CMRA along the trajectories (white arrow). The fluorescence was normalized to 1 at the opposite site of the synapse. The immunological synapse is indicated with “IS”. Cells with an actin response show a more than 2-fold higher fluorescent signal at the IS. Bars: 10µm. **(C)** Analysis of imaging flow cytometry images. First, a scatter plot of aspect ratio vs. area was used to gate for cells and exclude debris and control beads (R1). Second, a gradient RMS (root mean square for image sharpness) histogram was used to gate for cells in focus (Focus). Third, the intensity of PE/Cy7 (CD56) was gated against the intensity of Emerald-Lifeact (EmLA). Double positive events were categorized as conjugates and used for subsequent image analysis. We defined AR^+^ CLL cells as target cells with an increased relative intensity of EmLA fluorescence in the IS mask in relation to the total intensity of EmLA in target cells. For determination of granzyme B content in CLL cells, the NK cell part of the immune synapse is excluded from the analysis. **(D)** Immune synapse definition using IDEAS. Cell shape defined by surface labelling (CD56) or EmLA expression was extended by 3px in all dimensions using the dilate function. The overlapping region was defined as the immune synapse mask (IS mask) in NK cell-CLL cell conjugates. Created with BioRender.com

Supplementary Figure 2
**(A)** Imaging flow cytometry analysis of total F-actin fluorescent intensity in AR^+^ and AR^-^ HG-3, PGA-1, JVM-3, and MEC-1 cells. Differences between cell lines are the result of differences in transduction efficiency and transgene expression. Analysis was performed using a Wilcoxon paired t-test. **(B)** Apoptosis in HG-3 and PGA-1 conjugates with NK-92MI as evaluated by imaging flow cytometry. Cells were gated based on the absence (AR^-^) or presence (AR^+^) of an actin response. * denotes to p < 0.05, ** denotes to p < 0.01 **(C)** Measurement of SYTOX Blue fluorescence intensity over time in the MEC-1 cells with and without an actin response following conjugation with NK cells. Image J software was used to do the quantification of LSM880 acquired images. **(D)** Evaluation of GTP-loaded Cdc42 by G-LISA colorimetric activation assay. MEC-1 cells were pre-treated for 1 h with 50 µM ZCL278 before drug was removed and cells resuspended in fresh complete medium. Cells were allowed to recover for 1 and 5 hrs after drug removal before stimulation with 0.1 µg/mL human recombinant EGF for 15 minutes to measure inducible CDC42 activity. *** denotes to p < 0.0001. **(E)** Spontaneous cell death in HG-3, PGA-1, JVM-3, MEC-1 cells treated with either DMSO or 50 µM ZCL278 for 1 h. Drugs were removed and cells allowed to recover for 45 minutes before cell death analysis using Annexin V and propidium iodide.

Supplementary Figure 3
**(A)** Imaging flow cytometry analysis of DMSO- or ZCL278-treated HG-3 and PGA-1 with regards to actin response frequency in conjugates. **(B)** Cytotoxicity assays of DMSO- or ZCL278-treated HG-3 and PGA-1 cells with NK-92MI at E:T ratio of 1:1 and 5:1. **(C)** Apoptosis assay of DMSO- or ZCL278-treated HG-3 and PGA-1 cells with NK-92MI effector cells. Cells were co-cultured for 45 minutes before live/dead staining with Annexin V and propidium iodide. **(D)** Quantitative imaging flow cytometry analysis of granzyme B load in HG-3 and PGA-1 CLL cells in conjugation with NK-92MI cells. **(E)** Imaging flow cytometry analysis of granzyme B load in DMSO- or ZCL278-treated HG-3 and PGA-1 CLL cells after 45 minutes of co-culture with NK-92MI cells. **(F)** Flow cytometry gating strategy for buffy coat-derived PBMC before and after negative selection for NK cells.
