# Supplementary figures and images for: Intrinsic Resistance of Chronic Lymphocytic Leukemia Cells to NK Cell-Mediated Lysis Can Be Overcome In Vitro by Pharmacological Inhibition of Cdc42-Induced Actin Cytoskeleton Remodeling
Source: Front Immunol. 2021 May 24;12:619069. doi: 10.3389/fimmu.2021.619069 (PMC8181408; doi:10.3389/fimmu.2021.619069)

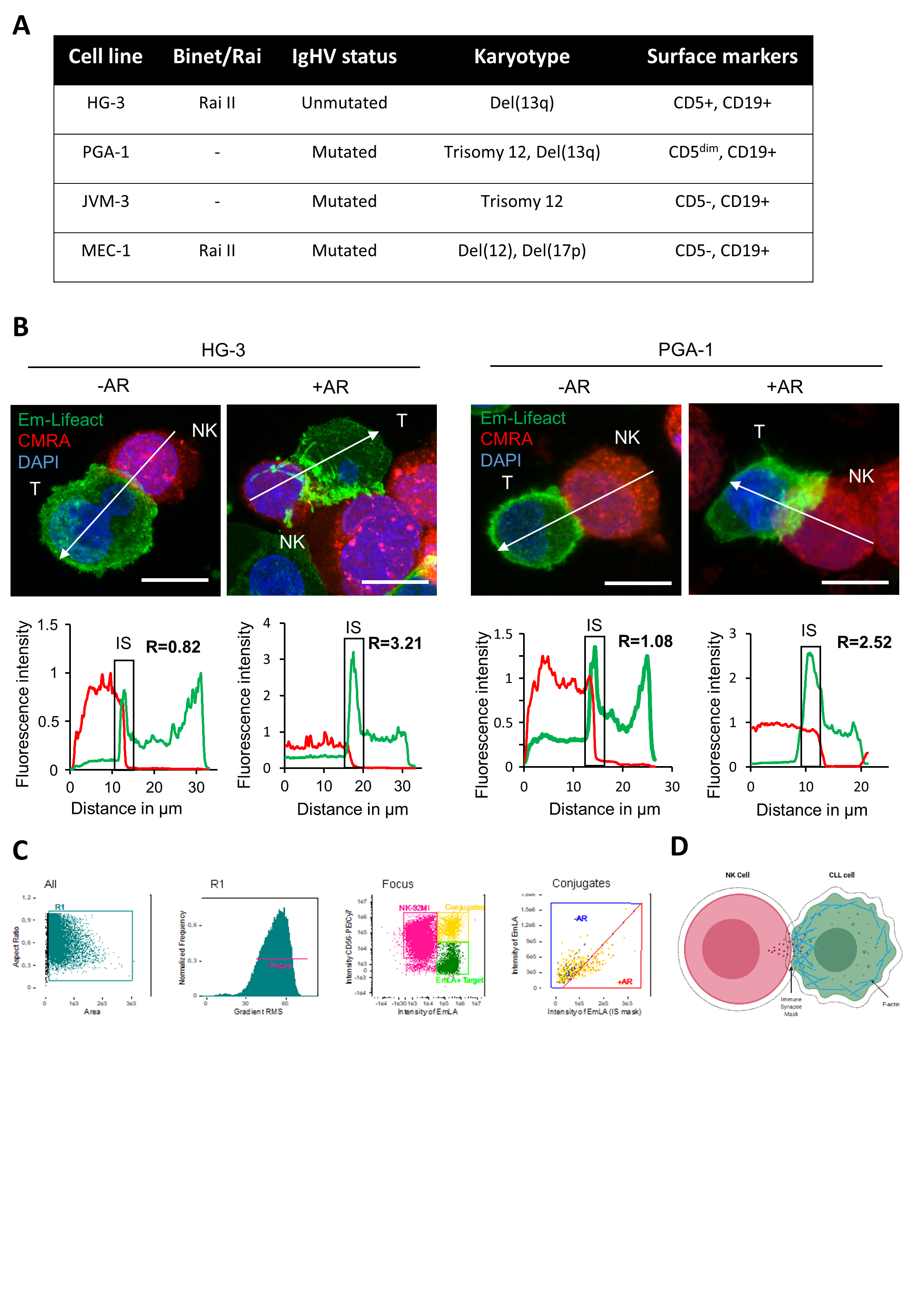

Supplement: Supplementary Figure 1 — (A) List of CLL cell line characteristics. (B) Confocal microscopy pictures of HG-3 and PGA-1 Emerald-Lifeact cells (T) in conjugation with NK-92MI cells (NK) with and without an actin response. Charts below show the relative fluorescent intensity of Emerald-Lifeact and CMRA along the trajectories (white arrow). The fluorescence was normalized to 1 at the opposite site of the synapse. The immunological synapse is indicated with “IS”. Cells with an actin response show a more than 2-fold higher fluorescent signal at the IS. Bars: 10µm. (C) Analysis of imaging flow cytometry images. First, a scatter plot of aspect ratio vs. area was used to gate for cells and exclude debris and control beads (R1). Second, a gradient RMS (root mean square for image sharpness) histogram was used to gate for cells in focus (Focus). Third, the intensity of PE/Cy7 (CD56) was gated against the intensity of Emerald-Lifeact (EmLA). Double positive events were categorized as conjugates and used for subsequent image analysis. We defined AR+ CLL cells as target cells with an increased relative intensity of EmLA fluorescence in the IS mask in relation to the total intensity of EmLA in target cells. For determination of granzyme B content in CLL cells, the NK cell part of the immune synapse is excluded from the analysis. (D) Immune synapse definition using IDEAS. Cell shape defined by surface labelling (CD56) or EmLA expression was extended by 3px in all dimensions using the dilate function. The overlapping region was defined as the immune synapse mask (IS mask) in NK cell-CLL cell conjugates. Created with BioRender.com [file Image_1.tif]

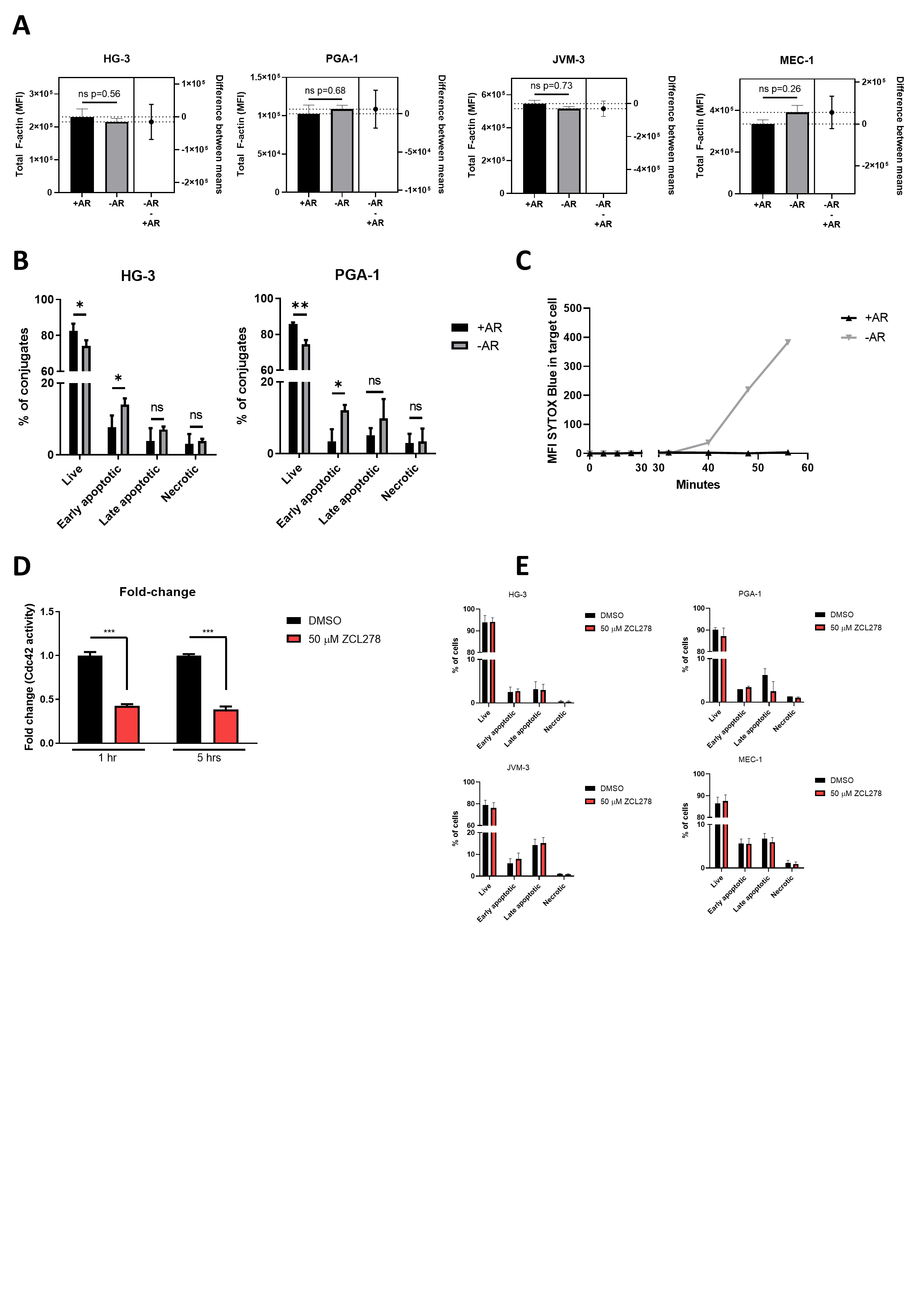

Supplement: Supplementary Figure 2 — (A) Imaging flow cytometry analysis of total F-actin fluorescent intensity in AR+ and AR- HG-3, PGA-1, JVM-3, and MEC-1 cells. Differences between cell lines are the result of differences in transduction efficiency and transgene expression. Analysis was performed using a Wilcoxon paired t-test. (B) Apoptosis in HG-3 and PGA-1 conjugates with NK-92MI as evaluated by imaging flow cytometry. Cells were gated based on the absence (AR-) or presence (AR+) of an actin response. * denotes to p < 0.05, ** denotes to p < 0.01 (C) Measurement of SYTOX Blue fluorescence intensity over time in the MEC-1 cells with and without an actin response following conjugation with NK cells. Image J software was used to do the quantification of LSM880 acquired images. (D) Evaluation of GTP-loaded Cdc42 by G-LISA colorimetric activation assay. MEC-1 cells were pre-treated for 1 h with 50 µM ZCL278 before drug was removed and cells resuspended in fresh complete medium. Cells were allowed to recover for 1 and 5 hrs after drug removal before stimulation with 0.1 µg/mL human recombinant EGF for 15 minutes to measure inducible CDC42 activity. *** denotes to p < 0.0001. (E) Spontaneous cell death in HG-3, PGA-1, JVM-3, MEC-1 cells treated with either DMSO or 50 µM ZCL278 for 1 h. Drugs were removed and cells allowed to recover for 45 minutes before cell death analysis using Annexin V and propidium iodide. [file Image_2.tif]

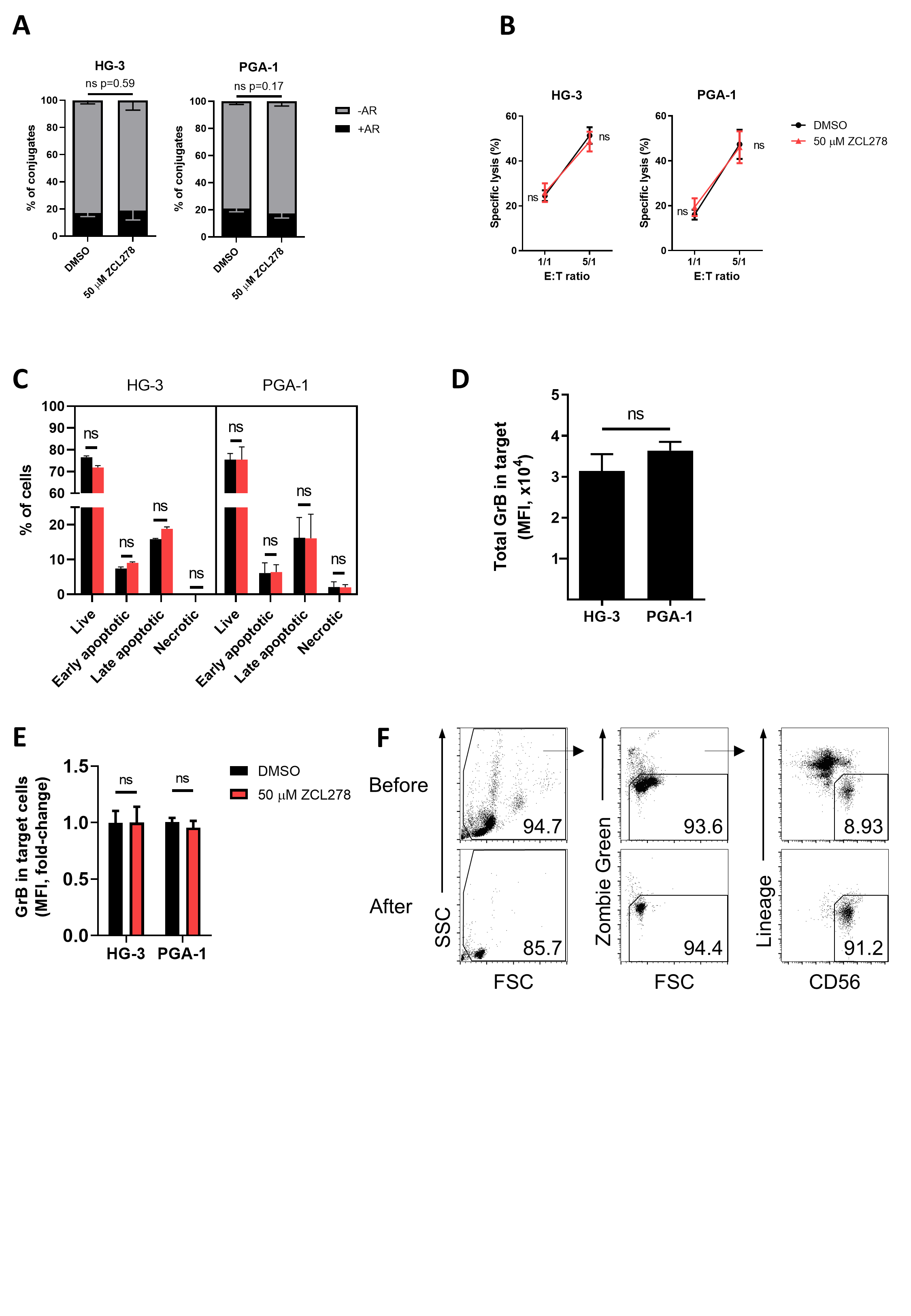

Supplement: Supplementary Figure 3 — (A) Imaging flow cytometry analysis of DMSO- or ZCL278-treated HG-3 and PGA-1 with regards to actin response frequency in conjugates. (B) Cytotoxicity assays of DMSO- or ZCL278-treated HG-3 and PGA-1 cells with NK-92MI at E:T ratio of 1:1 and 5:1. (C) Apoptosis assay of DMSO- or ZCL278-treated HG-3 and PGA-1 cells with NK-92MI effector cells. Cells were co-cultured for 45 minutes before live/dead staining with Annexin V and propidium iodide. (D) Quantitative imaging flow cytometry analysis of granzyme B load in HG-3 and PGA-1 CLL cells in conjugation with NK-92MI cells. (E) Imaging flow cytometry analysis of granzyme B load in DMSO- or ZCL278-treated HG-3 and PGA-1 CLL cells after 45 minutes of co-culture with NK-92MI cells. (F) Flow cytometry gating strategy for buffy coat-derived PBMC before and after negative selection for NK cells. [file Image_3.tif]
